# Time-of-Flight Microwave Camera

**DOI:** 10.1038/srep14709

**Published:** 2015-10-05

**Authors:** Gregory Charvat, Andrew Temme, Micha Feigin, Ramesh Raskar

**Affiliations:** 1 Massachusetts Institute of Technology Media Lab, 75 Amherst St., Cambridge, Massachusetts, USA, 02139; 2 Department of Electrical and Computer Engineering, Michigan State University, East Lansing, Michigan, USA 48824

## Abstract

Microwaves can penetrate many obstructions that are opaque at visible wavelengths, however microwave imaging is challenging due to resolution limits associated with relatively small apertures and unrecoverable “stealth” regions due to the specularity of most objects at microwave frequencies. We demonstrate a multispectral time-of-flight microwave imaging system which overcomes these challenges with a large passive aperture to improve lateral resolution, multiple illumination points with a data fusion method to reduce stealth regions, and a frequency modulated continuous wave (FMCW) receiver to achieve depth resolution. The camera captures images with a resolution of 1.5 degrees, multispectral images across the X frequency band (8 GHz–12 GHz), and a time resolution of 200 ps (6 cm optical path in free space). Images are taken of objects in free space as well as behind drywall and plywood. This architecture allows “camera-like” behavior from a microwave imaging system and is practical for imaging everyday objects in the microwave spectrum.

Microwaves (300 MHz to 30 GHz) have the ability to penetrate many materials which are opaque to visible light. In order to achieve diffraction-limited images with high resolution at long wavelengths, imaging systems must capture energy over a large aperture. Furthermore every-day objects and features that are not much larger than the illumination wavelength appear specular in microwave images, leading to unrecoverable surfaces that we refer to as stealth regions (see Fig. 1C).

Two common methods of microwave imaging are phased-array imaging and synthetic aperture imaging. These techniques require spatially sampling a large aperture at half a wavelength (λ/2) or higher density to avoid aliasing^1^. Phased array systems accomplish this with hundreds or thousands of phase-coherent elements which are phase-shifted to sample a volume in 3D^2^. Synthetic aperture techniques trade off element-count for measurement-time by scanning a single phase-coherent element across the aperture^2^,^3^,^4^. A hybrid system consisting of a synthetic aperture with switched arrays of coherent elements can provide high-resolution images, especially when electronic scanning is used^5^,^6^. Our work reduces measurements by sampling only in a focal plane, thus our system is neither a phased-array nor a synthetic aperture system.

We demonstrate a low-cost imaging solution that can complement or provide alternatives to costly phased array or synthetic aperture systems. Cost and complexity of imaging systems must be reduced below military or security budgets to allow for widespread adoption in commercial and consumer applications. In this work we present a proof-of-concept imaging system and data-processing chain that offer advantages over synthetic aperture and phased array systems, however many applications are still best serviced by these systems. Our imaging system consists of an illumination source, a dish reflector, and a sensor in the focal plane of the reflector. The reflector reduces the size of the sensing area, and reduces system cost and complexity. In our implementation, we scan a single sensor across the focal plane. Creation of a solid-state focal plane array can be difficult; however, this has been accomplished by others at comparable frequencies^1^,^5^,^6^,^7^.

Our system is a departure from traditional radar imaging systems and does not fall into any one category. We do not cover or sample the entire aperture, in order to reduce complexity, computing needs, and the number of sample measurements.

Covering an aperture with active elements is costly due to components, calibration, and synchronization. For example, in order for a phased-array system to theoretically achieve an angular resolution of 0.5 degrees at 12 GHz without aliasing, an array of 7,482 active-sensors would need to be placed across a circular aperture with diameter of 1.22 m. Other works have reduced the number of measurements necessary by employing sparse reconstruction, passive metamaterial apertures, or multiple lenses^6^,^7^,^8^,^9^,^10^,^11^,^12^,^13^. Our method samples the focal plane of a simple passive reflector, which allows us to trade-off field-of-view (FOV) for a reduction in the number of measurements for imaging on dense scenes. Our method covers the same aperture with a passive reflector, enabling the camera to achieve 0.5 degree resolution. By limiting the field of view to objects in front of the camera, our method uses only 1,681 measurements (fewer than 1/4^th^ of the measurements required for an equivalent phased array) while imaging at the same resolution. The measured PSF of our system is 1.5 degrees (larger than the theoretical 0.5 degrees) due to aberrations in the parabolic reflector and limitations in isolating individual wavelengths without trading-off time resolution. While sampling a larger aperture may produce higher resolution images, one increases sampling time and data size.

By sampling a focal plane like a traditional camera, we allow the use of computational photography techniques such as compressed sensing or coded apertures^14^,^15^. The prototype system does not capture all pixels simultaneously like a film camera; instead it operates as a composite camera like a gigapixel camera^16^, DARPA’s ARGUS-IS camera^17^ or a femtosecond camera^18^.

We demonstrate a microwave camera capable of producing images that recover 2D brightness, depth, and multi-spectral response of objects in the microwave regime. This is shown for objects in the open, behind drywall, and behind plywood (Fig. 2). We also show movies of transient microwave propagation (Movie S1), which have attracted interest in a variety of communities^18^,^19^,^20^,^21^,^22^. This transient response is calculated based on spatial and frequency sampling as described below. Furthermore we exploit the 3D capabilities of the camera to segment parts of the scene and recover images of multi-layered objects within containers (Fig. 3). Our system exhibits many of the behaviors of a visible-light camera, including depth of field; this can be used to reduce the impact of background-clutter outside of the focus area.

Images in the microwave spectrum can be captured with this camera through partitions such as dry-wall and plywood. An image of a mannequin behind obstructions is shown in Fig. 2E,F. The mannequin was placed approximately 2.1 m (7 ft) in front of the imaging system and the partition approximately 15 cm–30 cm (6 in to 12 in) in front of the mannequin. The plastic mannequin was covered in aluminum foil to increase reflectivity and as a rough approximation of the human body^23^,^24^ since plastic mannequins are transparent. Rubber could be used to better represent the body in future images.

In order to image objects behind partitions, we exploited the transparency and low-reflectivity of common materials in the X-band^25^. We note that the reflection from our target (mannequin) was much stronger than the reflection from the wall. Any large reflections from the partition may be ignored by looking at the image in the temporal domain, i.e. frame-by-frame for the time-of-flight movie. We are able to time-gate the frames with the reflection from the partition.

The images in Fig. 2D–F were captured using a single illumination source and thus suffer from stealth regions due to the specular reflections off of non-cooperatively oriented surfaces at microwave frequencies and shadowing.

Another challenge of imaging objects in the microwave spectrum is the specular nature of reflections off of surfaces with features sizes that are smaller than the illumination wavelength. This leads to images with gaps, blind-spots, or invisible objects. We introduce multiple points of illumination and a projective recombination of the illuminations to capture more information from every point in the scene, thus forming a more complete microwave image. Some of these issues have been discussed in the literature with regards to synthetic aperture radar (SAR) imaging systems^1^,^5^,^7^,^26^. The work presented here is at longer wavelengths than the previous work, and benefits from advances in computational photography^27^.

To address these shortcomings, a method of recovery was implemented using multiple illumination sources. Multiple data cubes were captured, each for a different illumination position. The data cubes were corrected for the projection geometry of each transmitter/receiver position so that reflections in the data cube are aligned with a voxelized Cartesian space of the scene. The data cubes are then fused by finding the maximum amplitude of the scattered reflection at each voxel. Using this methodology, an unobscured foil-covered mannequin was imaged from three different points of illumination (Fig. 1B), revealing regions such as the arms, head, and torso.

The camera evaluated in this report operates in the X-band region (8–12 GHz) of the electromagnetic spectrum using a frequency-modulated continuous-wave (FMCW) transmitter/receiver.

A detailed description of the camera, its individual parts, and the design space is presented in the supplementary material. The transmitter (Fig. 1C) is re-locatable and enables the scene to be illuminated from arbitrary locations. Objects in the scene reflect the transmitted waves, which are then collected and focused by a parabolic dish onto the focal plane. The parabolic reflector has a diameter of 1.22 m and a focal length of 0.45 m. A sensor is raster scanned in X and Y through the focal plane of the dish and a 41 pixel x 41 pixel image is captured over a 25.4 cm by 25.4  cm area. At each measurement point, the emitter is linearly swept from 7.835 GHz to 12.817 GHz over a 10 ms period, and the received signal is demodulated by mixing the received signal with the transmitted signal and then applying a low pass filter. The amplitude of the demodulated signal is sampled at 200 kHz using an analog-to-digital converter. The system is calibrated by measuring the response of an aluminum sphere placed in front of the system. The scanning system and receiving element supports are covered in radar absorbing material to minimize interference effects, furthermore they are outside of the depth of field of the camera. Each scan leads to a data-cube whose axes are angle in the horizontal direction, angle in the elevation direction, and the microwave time-of-flight in the third dimension (*θ*, *ϕ*, *t*). Time-of-flight data is computed based upon FMCW radar processing^28^. The demonstrated system has a Rayleigh-limited depth resolution of 200 ps, thus facilitating visualization of physical electromagnetic phenomena such as pulse reflections of microwave energy as a function of time propagating through an image.

We validate the system capabilities with a single illumination point before showing the benefit of multi-point illumination. With the system, we visualize microwaves interacting with a scene, or “microwaves-in-flight” movies at a sampling period of 200 ps (6 cm). A color-coded time sequence from a visualization of microwaves passing over a metal peacock ornament is shown in Fig. 3A. The energy front travels from the right side of the image and propagates to the left.

The illumination bandwidth of the camera can be exploited to generate multi-spectral images from a single data-set. In order to demonstrate this, we captured an image of a set of wires of decreasing length. The longest wires reflect over the entire bandwidth, while the shortest wires reflect only the shorter wavelengths. One can see in Fig. 4C that the extracted frequency-mapped color of each point varies with resonator size. The shortest wires are mostly blue as they do not appear in the lower (red) frequency band. The mapping between colors and frequencies as well as the trade-off between depth-resolution and spectral resolution is shown in the supplementary materials. Validation of the ability of the system to resolve the depth of an object in a scene is also presented in the supplementary materials.

Through multi-spectral imaging, we can observe variation of images across a wide set of wavelengths (2.5 cm–4 cm). The mannequin appears different across this frequency range (Fig. 1E) due to a varying point spread function (PSF) and reflectance properties at different illumination frequencies. This can be exploited to classify materials in scenes.

One practical application of this camera is the nondestructive testing or evaluation of an object in a container in order to ensure its structural health or topology. A demonstration of this is shown for a cardboard shipping box in which the letters “M,” “I,” and “T” are constructed out of point targets with each letter mounted to a separate layer of microwave-transparent styrofoam (Fig. 3B,C). By time-gating the data cube in software, it is possible to separate each layer placed along an axial plane inside the box.

The current camera utilizes a large reflector (1.22 meters), and the image is captured by mechanically scanning the focal plane which requires a one-hour long scan. The receiver is built using off-the-shelf hardware, although conventional network analyzers and radar systems can also work as a transmitter/receiver. Real-time imaging can be realized using a reconfigurable focal-plane sensor and sparse recovery^7^,^11^,^13^, or utilizing multiple receivers, such as the recent CMOS chip realizations^29^. Using a shorter wavelength of transmission can reduce the size of the reflector.

More elaborate processing of multi-illumination data can be exploited to recover even more information from the scene, such as the alignment of the surface-normals and improved 3D reconstructions. Multi-illumination can be used to extract surface normal orientation in the scene to improve 3D reconstruction^30^. Furthermore, the dependency of the reflectivity of a scene on the illumination wavelength and incoming angle can be used to identify the material properties of objects in the scene or reduce the number of illumination points necessary to capture a complete image.

The architecture demonstrated in this paper is a less complex approach to forming radar images of everyday objects. In addition, the image formation model can be more easily adopted into computer-vision algorithms and is a practical step towards scene-understanding in microwave-imaging. The multi-illumination architecture may be extended to address issues in long-wavelength imaging in other modalities such as acoustics, microscopy, and other RF applications (Wi-Fi imaging or automotive radar). Furthermore, by varying the distance between the sensor array and the reflector, one can dynamically change the focused-zone of the system and reduce the response of a known-clutter region. The microwave camera has applications in the recovery of survivors in disaster situations, imaging in hazardous conditions, and non-destructive testing.

## Additional Information

**How to cite this article**: Charvat, G. *et al.* Time-of-Flight Microwave Camera. *Sci. Rep.* **5**, 14709; doi: 10.1038/srep14709 (2015).

## Supplementary Material

Supplementary Information

Supplementary Video

## Figures and Tables

**Figure 1:**
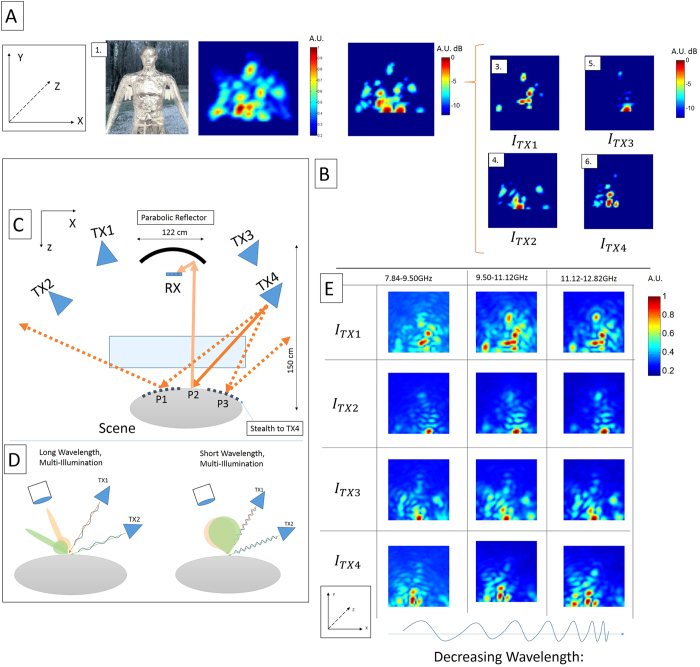
Can we recover diffuse-like images at microwave wavelengths? Combining images from multiple illumination sources creates microwave images with fewer stealth regions. In (**A**) we see a visible-light image (**A1**) and a microwave image of an unobscured mannequin (**A2, A3**) generated by projective recombination of illumination images (**B1–4**). In (**C**) the transmitters are on the left and right of the parabolic reflector. Incident rays from TX4 reflect off of P1 and P3, and never return to the camera; however, the reflection from P2 does return and is visible to the camera. Introducing other illumination points allows P1 and P3 to be visible to the camera. In (**D**) the reflectance lobes for short wavelengths are wider than the reflectance lobes at long wavelengths^31^, thus the multi-spectral images of the scene provide additional information depending on the size of features in the scene. In (**E**) each image is broken down into the energy received from three spectral bands, leading to diversity in reflectance properties.

**Figure 2:**
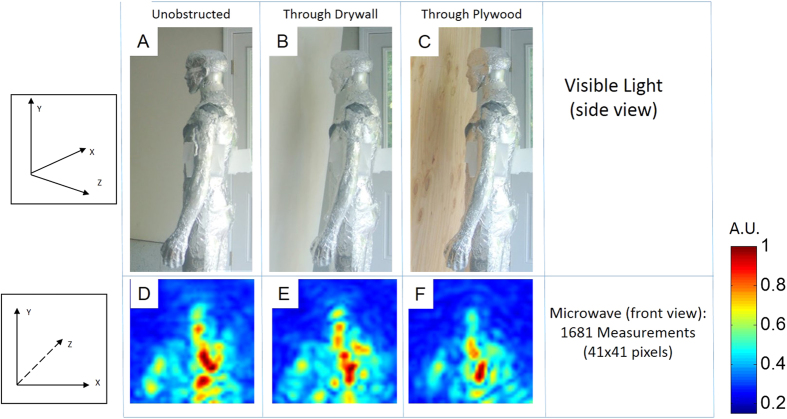
The microwave camera can image at wavelengths which easily penetrate drywall and plywood. In (**A**–**C**) an image is taken of a mannequin wrapped in aluminum foil in free-space, placed behind 12.7 mm thick dry-wall, and behind 11.9 mm thick plywood. The mannequin is wrapped in foil in order to approximate the strong reflectivity of the human body^23^. The recovered 41 pixel by 41 pixel microwave-photographs are shown below each visible-light image (**D–F**).

**Figure 3:**
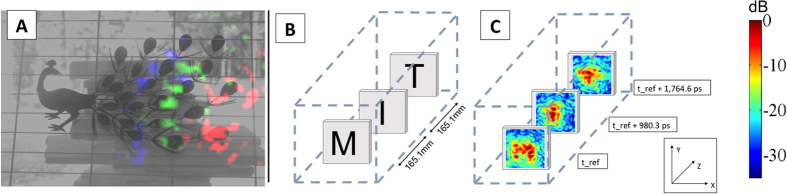
Two practical applications of a time-of-flight microwave camera are demonstrated. In (**A**) we visualize the propagation of microwaves across a metal peacock ornament as a color-coded time sequence, similar to a “light in flight” movie^18^,^22^. Here a grayscale, visible-light image is overlaid with color-coded data from the microwave camera. The red channel is the response at an early reference time, the green channel is the response at an additional 588.2 ps, and the blue channel is the response 1078.4 ps after the reference time. One can see the curve of the microwave as it crosses the scene and reflects off of features. In (**B**) the microwave camera is used to inspect the contents of a box to ensure proper packaging. A set of push pins are placed on three pieces of styrofoam inside of a shipping box in the shape of the letters “M”, “I”, and “T”. By separating the images in time, it is possible to see the three different layers. In (**C**) the average intensity at 700 ps around each center point is shown.

**Figure 4:**
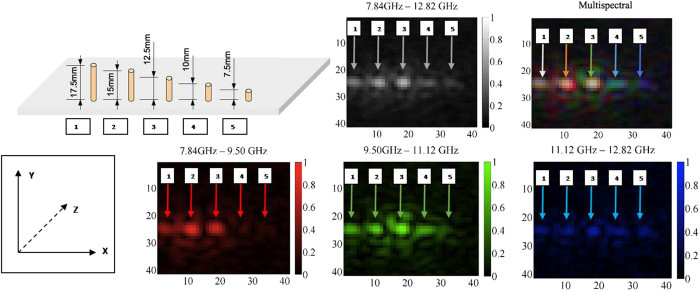
The illumination bandwidth can be exploited to generate multispectral microwave images. The reflectance properties of five sub-wavelength wire resonators of decreasing length are shown. In (**A**) there are five wires of decreasing length (L to R: 17.5 mm, 15 mm, 12.5 mm, 10 mm, and 7.5 mm) placed vertically in styrofoam (which is transparent in the X-band). In (**B**) a gray scale linear intensity image is shown (full 5 GHz bandwidth). In (**C**) a multi-spectral image is shown where the primary colors red, green, and blue represent the lower, middle, and upper frequency bands of illumination, respectively. The smaller wires are not as reflective of the longer wavelengths, causing them to appear bluer. The individual frequency band images are shown in (**D–F**).
