# Supplementary material for: Time-of-Flight Microwave Camera
Source: Sci Rep. 2015 Oct 5;5:14709. doi: 10.1038/srep14709 (PMC4592950; doi:10.1038/srep14709)
Supplement: Supplementary Information [file srep14709-s1.pdf]

Time-of-Flight Microwave Camera

Gregory Charvat,[1*] Andrew Temme,[2]
Micha Feigin,[1] Ramesh Raskar[1]
[1]Massachusetts Institute of Technology Media Lab,
75 Amherst St., Cambridge, Massachusetts, USA, 02139
[2]Department of Electrical and Computer Engineering,
Michigan State University, East Lansing, Michigan, USA 48824
[*]To whom correspondence should be addressed; E-mail: charvatg@gmail.com

Supplementary Materials

- Additional Related Work
- Focal-length versus Frequency Discussion
- Angular Resolution of the System
- Materials and Methods
- Movie 1
- External Database S1
- Figures S1-S11
- Table S1

Additional Related Work

Please see Table 1 for a numerical performance comparison of this time-of-flight microwave camera and other related work.

Phased Arrays and Synthetic Aperture Radar Work began on phased array radars in the 1960's as a method to dynamically control the beam direction of a radar antenna [1]. Phased-arrays have the advantage of a large

field-of-view and fast scans, however they require many phase-coherent elements to form an image [1]. Phased array systems on aircraft early warning radars were composed of thousands of phase-controlled elements. Synthetic aperture systems form large radar apertures by scanning a single phase-coherent element across an aperture. This has been used for NASA's shuttle radar topography mission to achieve a spatial resolution of 5-10m by sampling an aperture of 50km at a density of $\lambda/2$ in order to avoid aliasing [2]. Our methodology does not sample the camera aperture with active elements, and instead captures reflected waves using a passive parabolic reflector and concentrates the active elements to a smaller focal plane. This allows for high resolution imaging with fewer measurements required to form an aliasing-free image ($< 1/4$ of the measurements), however at a limited field of view and depth of field.

Focal Plane Array Optical imaging techniques have also been applied to millimeter wave (mmW) and THz radio telescopes, where focal plane arrays (FPAs) are placed at the focal point of a parabolic reflector. A wideband 86 GHz–115 GHz FPA was developed for the 14 m radio telescope at the Fife College Radio Astronomy Observatory (FCRAO) consisting of 15 elements [3, 4]. FPAs for 1.3 mm and 3 mm were also designed for the 12 m radio telescope at the National Radio Astronomy Observatory (NRAO) and the 14 m radio telescope at FCRAO [5]. A 160 element FPA bolometer for the 10 m radio telescope in the south pole, as presented in [6], was developed to operate at 90 GHz, 150 GHz, and 220 GHz. A high resolution map of cosmic background radiation was acquired by the Plank radio telescope satellite. The lower frequency FPAs for 30 GHz and 70 GHz and the optical systems were described in [7]. Additional FPAs at mmW bands were developed to measure cosmic background radiation polarization [8] and for 230 GHz radio astronomy applications [9]. At microwave frequencies, [10] proposed and modeled an FPA concept for 18 GHz–26.5 GHz using an array of Vivaldi antenna elements. These are radio telescopes where the instrument is focused at infinity and a limited number of pixels are used. Advanced focal plane array concepts have been developed for THz frequency ranges, such as the 1.6 THz FPA proof-of-concept discussed in [11]. To achieve mmW performance using 65 nm CMOS technology, [12] presents an interesting approach for a 200 GHz, 4-by-4 element FPA implemented using a regenerative receiver architecture. Other work with 230 GHz general purpose FPAs is ongoing [13].

Computational Computational approaches have been applied to microwave imaging for through-wall imaging, non-destructive testing, and human interaction. Adib et al. show in [14, 15] that a set of multiple-input-multiple-output (MIMO) Wi-Fi radios can be used to localize reflectors through walls and track the reflectors' position. Their work uses adaptive array processing to null the first flash from the wall. Charvat et al. [16], use a MIMO radar system to localize targets through walls and concrete, and an analog filter to reduce the flash from the wall. Our system surpasses these systems in being able to return a full X-Y image with 3D shape information to the user. Ghasr et al. demonstrated a microwave camera at 24 GHz with a PIN-diode based slot-array multiplexer [17]. Our method achieves images at better angular resolution with a fewer number of measurements due to the large passive-aperture in our method.

There has also been work in reducing the number of measurements required to perform imaging through compressed sensing. Spinoulas et al. introduce a lens and mask based sparse capture/reconstruction for passive millimeter wave imaging [18]. Chan et al. proved compressed sensing could be used in the terahertz regime [19]. We extend this work to active, coherent microwave imaging which means our architecture can address sparse measurements in both X-Y and in depth. Cull et al. demonstrate compressive millimeter wave holography by subsampling and reconstructing a Gabor hologram [20].

Hunt et al. show that a metamaterial aperture with a frequency dependent aperture function can be used to image metal targets, and demonstrate using a retroreflective corner cube [21]. They further show that the number of measurements can be reduced through sparse reconstruction. While their work addresses many of the complications for consumer radar imaging systems, our work does not trade-off angular sampling for frequency, and enables 3D radar imaging with spatially-independent spectral information, allowing the user to arbitrarily break down an image by its spectral information at no loss of X-Y resolution. This has also been accomplished using dynamic metamaterial apertures [22]. Our architecture is able to incorporate multi-spectral capabilities while still providing depth, 3D shape, and a reduced number of sensor elements.

Focal-length versus Frequency

The focal point (spatially) for an ideal reflector will not change with frequency, however the antenna gain will increase as the wavelength decreases

(see Figure S1). Conducting parabolic reflectors (not lenses) at these wavelengths/bandwidth show negligible dispersion characteristics. In measuring our setup, we achieved close to the resolution that we expected. For those reasons, we assume that our setup approximates a reflective parabolic mirror. Furthermore, changes in antenna gain due to frequency are equalized during our calibration procedure.

Angular Resolution of the System

We note that the diffraction-limited resolution of our system should be

$$\theta = 1.22\lambda/D \tag{1}$$

where D is the aperture of the system (1.22 meters). The angular resolution thus depends on wavelength.

In order to measure the PSF of the system, we used 1/4 in radius spheres placed at a distance of 2.06 m in front of our camera, thus each sphere covered 0.35 degrees (much below the resolution of our system). Thus each sphere can be considered a point source for the PSF. The first row of spheres were spaced apart so that the camera viewed them as separated by 3.5 degrees.

Figure S2 shows the target setup (with point reflectors) on the left. On the right, you can see a reconstruction which is corrected for the camera projection, with the correct positions overlaid on top in red circles. Note that this is a horizontal view of 3D data (X,Y).

By capturing the intensity along the X dimension at various frequencies we can show the width of the PSF. Here we show the width of the PSF at a wavelength of 2.56 cm to be: 1.512 degrees. Aberrations in our parabolic reflector and positional error in measurements can lead to a wider PSF than expected.

At higher frequencies, this PSF is smaller, however the image SNR increases due to the bandwidth limitations of our electronics. A higher center frequency will lead to a tighter PSF. In the revision, we address this by stating that our system can achieve a theoretical resolution of 0.5 degrees, given a perfect reflector and sufficient system bandwidth.

Materials and Methods

The experimental setup is shown in Fig. S3. Akin to a flash for an optical camera, this work uses an antenna (ANT1 in Fig. S4) to illuminate the scene. This microwave camera uses an ultra-wideband (UWB) antenna emitting a continuous wave (CW) of which the frequency is linearly swept from 7.835 GHz to 12.817 GHz, for a bandwidth, BW, of 4.982 GHz, over a chirp width, T_p, of 10 ms. This is represented by

$$TX(t) = \cos\big(2\pi(f_{osc} + \frac{c_r}{2}t)t\big), \tag{2}$$

where f_{osc} is the start frequency of the linear-ramp modulated voltage controlled oscillator (VCO) and c_r is equal to the radar chirp rate BW/T_p.

Scattered microwaves are collected and focused by a lens-equivalent dish of radius r. Typically a parabolic reflector focuses objects in the far field to the focal point $f = r/2$. In this paper the target scene is in the near-field of the dish and is focused to a point between r and $r/2$ (Fig. S5). Using a parabolic reflector is less expensive and less difficult to mount than using an equivalently sized dielectric lens.

An X band (WR-90) waveguide probe (ANT2) is mounted to an X-Y translation stage and is used to sample the fields over the image plane. Scattered and focused signals are collected by ANT2 and are represented by (without loss of generality, ignoring amplitude coefficients)

$$RX(t) = TX(t - t_{delay}), \tag{3}$$

Roundtrip time t_{delay} is defined as time taken to reach and scatter off of the target, return to and be focused by the dish, and be collected by the waveguide probe (ANT2).

The probe signal is fed into the receive port of a UWB frequency-modulated continuous-wave (FMCW) receiver [23]. The received delayed FMCW signal from ANT1 is then amplified by a low-noise amplifier (LNA1) then fed into a frequency mixer (MXR1). The illumination chirp is also fed into MXR1 which multiplies the sampled chirp by the reflected chirp,

$$m(t) = TX(t) \cdot TX(t - t_{delay}).$$

This product is amplified by the video amplifier and then digitized. This analog signal is digitized at a rate of 200 kHz.

The high frequency term of this cosine multiplication is rejected by the low-pass filter within the video amplifier resulting in

$$V(t) = \cos\big(2\pi c_r f_{osc} t_{delay} t + \phi\big). \tag{4}$$

When there is only one target present $V(t)$ is a sinusoidal wave with frequency proportional to t_{delay}; if multiple targets are present then $V(t)$ is a superposition of numerous sinusoids, each with frequencies proportional to the round-trip delay to its corresponding reflector.

A cube of data (angle, angle, time) is acquired using an X-Y translation stage where a 41 pixel by 41 pixel image is sampled. At each position 2000 analog samples are acquired and synchronized to the chirp time, T_p. This results in the signal $V(x_n, y_n, t)$, where x_n and y_n are the horizontal and vertical positions, respectively, of the waveguide probe, ANT2, in the 2D detector sampling plane.

To process and generate a microwave image, the time-average intensity of the entire signal $V(t)$ is computed for each pixel x_n and y_n, resulting in the image $s_{image}(x_n, y_n)$.

Calibration, Time, and Color

Calibration In order to calibrate the system's frequency response, we acquired data with a baseline target, an aluminum sphere placed in front of the camera, and performed background subtraction. We compared the measurement with the sphere with the Mie series solution for an ideal sphere of the same size [24]. The system phase and frequency response is calibrated by dividing the Mie data cube S_{Mie} by the calibration image S_{cal} :

$$S_{cf}(V(t)) = \frac{S_{Mie}(V(t))}{S_{cal}(V(t))}.$$

To apply calibration, each sample over $V(t)$ in the data cube is multiplied by the calibration factor S_{cf}, resulting in the calibrated data cube $S_{calibrated}\big(x_n, y_n, V(t)\big)$.

Time Domain To convert to the range domain after calibration is applied to $V(t)$, we observe the magnitude of the Fourier transform (DFT) at each pixel, resulting in $S\big(x_n, y_n, t_{delay}\big)$. We note that the time/frequency resolution of the system is limited by the bandwidth of the chirp.

Multi-Spectral The spatial frequency domain data cube is divided up evenly into three sub-bands over $V(t)$ of 666 samples each. These bands are color coded as red, green, and blue with mean center frequencies of red (8.66 GHz), green (10.32 GHz), and blue (11.97 GHz) respectively. To do this, $s\big(x_n, y_n, V(t)\big)$ becomes $s_{red}\big(x_n, y_n, V_{red}(t)\big)$, $s_{green}\big(x_n, y_n, V_{green}(t)\big)$,

and $s_{blue}\big(x_n, y_n, V_{blue}(t)\big)$. A DFT is applied to each sub-band in order to provide the range domain response of each color. The imagery is displayed in both range and color. Note that since FMCW processing uses system bandwidth to determine depth, each spectral division reduces the range domain resolution of the system. The reduction in range domain resolution is linearly related to the number of sub-bands desired in the final image.

An additional calibration step is taken when imaging in multi-spectral mode, white balance. White balance is achieved by imaging a relatively large (i.e. compared to a wavelength) sphere and scaling all color bands to the same amplitude as the center of the image. White balance is similarly applied to all multi-spectral imagery thereafter. The color image of a 76.2 mm diameter sphere in Fig. S6 shows the response of the broadband reflection of a sphere after white balance correction.

Design Space

The camera described in this paper enables us to view the world at microwave frequencies by combining optical design with time-resolved microwave techniques. The current implementation over samples the diffraction-limited image.

The Fresnel number for our proposed system at the largest wavelength is roughly $F \approx 20$. Since $F \gg 1$, Fresnel diffraction was used to model wave propagation of the imaging system. Geometric optics takes advantage of the small wavelengths of the relatively short wavelengths of visible light to make ray approximations for light propagation. However, at microwave and millimeter wavelengths the system design is more likely to run into the diffraction limit, thus it is important to consider pixel spacing, aperture size, and depth of field.

We follow the derivation of [25] and begin by deriving an expression of the wave field reflected off the scene measured at the image plane using the Fresnel diffraction integral

$$U_i(u,v) = \int_{-\infty}^{\infty}\int_{-\infty}^{\infty} h(u,v,\xi,\eta)U_0(\xi,\eta)d\xi d\eta \qquad (5)$$

and considering a point source at ξ , η (see Fig. S7)

$$\begin{aligned} h(u,v,\xi,\eta) &= -\frac{1}{j\lambda^2 d_i d_o} \\ \times \exp&\left(-j\frac{k}{2d_i}(u^2+v^2)+j\frac{k}{2d_o}(\xi^2+\eta^2)\right) \\ \times &\int_{-\infty}^{\infty}\int_{-\infty}^{\infty} P(x,y)e^{Ax^2+By^2+Cx+Dy}dxdy \end{aligned} \tag{6}$$

$$\begin{aligned} A &= j\frac{k}{2}(1/d_i+1/d_o-1/f) \\ B &= j\frac{k}{2}(1/d_i+1/d_o-1/f) \\ C &= -2j\frac{k}{2}(\xi/d_o+u/d_i) \\ D &= -2j\frac{k}{2}(\eta/d_o+v/d_i), \end{aligned} \tag{7}$$

where $P(x,y)$ is the 2D aperture function in the lens plane. The imaging equation $1/f = 1/d_o + 1/d_i$ makes the quadratic terms A and B go to zero. By introducing a magnification term $M = -d_i/d_o$ and the change of bases $\hat{\xi} = M\xi$ and $\hat{\eta} = M\eta$, the wave at the image plane can be expressed by the following convolution:

$$\begin{aligned} U_i(u,v) = \int\int\Bigg[&\hat{h}(u-\hat{\xi},v-\hat{\eta})\frac{1}{|M|} \\ &\times U_0\left(\frac{\hat{\xi}}{M},\frac{\hat{\eta}}{M}\right)\Bigg]d\hat{\xi}d\hat{\eta}, \end{aligned} \tag{8}$$

where:

$$\hat{h}(a,b) = 1/|M|\int\int P(x,y)e^{-j2\pi(a\hat{x}+b\hat{y})d\hat{x}\hat{y}}. \tag{9}$$

Thus the point spread function (PSF) can be expressed as

$$PSF(u,v) \propto \int\int P(x,y)e^{-j2\pi(u\hat{x}+v\hat{y})}d\hat{x}d\hat{y} \tag{10}$$

When $P(x,y)$ is a disk the size of the reflector, the solution for the PSF is the sombrero function resulting in an Airy disc. The Raleigh resolution limit of the system is defined by the first zero of the Airy disc which is

$$\triangle u = 1.22\lambda/D \tag{11}$$

where D is the diameter of the parabolic reflector.

The Airy disc defines the diffraction limited pixel pitch of the system and also its depth of field (DOF). Objects closer than the object plane will be focused to a point behind the image plane and thus will cast a circle of confusion on the image plane (Fig. S8). Similarly objects farther than the object plane will cast a circle of confusion. If the circle of confusion is smaller than the pixel pitch of the system, all scattering points within the depth of field will be in focus. Having a large depth of field is desirable for depth-imaging using the microwave camera system; however, a shallow depth of field has the advantage of allowing the system to focus-through clutter. In this derivation, the pixel pitch is placed at the diffraction limit of the system.

It is important to note that many approximations are made when using a parabolic reflector for imaging. The expression derived above through Fresnel-diffraction relies on the paraxial approximation in order to analytically solve the diffraction integral. In order to develop a more precise solution, we simulated Fresnel-Kirchhoff diffraction of the system to determine the focusing properties of the system at microwave wavelengths. An example of these simulations is shown in Fig. S7 and S9. While a parabolic reflector approximates a perfect lens, it suffers from off-axis aberrations such as comma for all points off-axis. Furthermore, while the focal point of a parabolic reflector is well-understood for point sources in the far-field, the focal length of the reflector deviates from the lens-law as the point source comes closer to the parabola.

When imaging a scene using a lens a practical concern is the depth of field of the system since scene reflectors out of the depth of field will have larger PSFs. Here we define the frequency dependent depth of field of the system. The depth of field as defined by the lens-law, PSF, and wavelength dependent circle of confusion, is expressed below.

The near focus of the system is determined using the lens law and assuming that pixels are spaced at the diffraction limit. This allows us to calculate a circle of confusion and determine the near and far focus of the system for a given focal length and wavelength.

$$O_{near} = \frac{1}{\frac{1}{f} - \frac{1}{I_N}} \tag{12}$$

$$O_{near} = \frac{fI}{I - f(\frac{1.22\lambda}{D^2} - 1)} \tag{13}$$

Here f is the focal length of the system, I is the distance between the

aperture and the sensor, D is diameter of the parabolic reflector (Fig S8).

$$O_{far} = \frac{1}{\frac{1}{f} - \frac{1}{I_F}} \tag{14}$$

$$O_{far} = \frac{f(\frac{1.22\lambda}{D^2} + 1)}{\frac{1.22\lambda}{D^2} + 1 - I} \tag{15}$$

This is the expression of the far focus, found by calculating the object distance which would cause a circle of confusion on the sensor of equivalent size to the diffraction limited pixel pitch. Subtracting these two equations results in a depth of field of the system:

$$DOF = O_f - O_n \tag{16}$$

Fig. S10 shows that for any given wavelength λ, the DOF is inversely proportional to the diameter increases. Intuitively, a larger aperture leads to a shallower depth of field. There is a point, however, where the DOF becomes infinite defined by $f = 1/(1 + 1.22\lambda I/d^2)$. At this point the DOF is long and the system is able to focus on many depths within the scene. At this point though, both the distance between the scene and the camera, and the pixel pitch (size of the receiving array) becomes large.

In order to achieve the maximum DOF for a diffraction limited system at a given pixel-pitch, one should select the largest focal length possible for the reflecting optics; however, this comes at the cost of increasing the object distance.

Knowing the DOF and out-of-field PSF is important for future applications involving model-based reconstruction through deconvolution such as that shown in [26, 27]. In order to theoretically determine the effect of focal-displacement on PSF, we show the ray-optics theoretical curve, a simulated wave-optics theoretical curve, and an experimentally measured curve in Fig. S11. This information is important to understand imaging performance outside of the system's DOF.

The performance of the camera and a comparison to lens-based and lensless cameras are shown in Table S1.

Additional Results

Results in addition to those discussed in the main paper are presented here for completeness.

Grid Fly Through A frame by frame "fly-through" of a grid of spheres is shown in Fig. S12. The grid is shown in Fig. S13. This demonstrates the capability of the camera to separate a set of 15 reflectors into five different depth planes. This also demonstrates that despite shadowing, the reflectors are still visible. Furthermore it can be seen that reflectors in the back-row are closer to the optical axis (center pixel) than the front row. This is due to the camera projection.

Movie S1

Movie S1 shows the system and time-of-flight capabilities.

External Database S1

Please find code and data available at http://scripts.mit.edu/MicrowavePackage.zip/

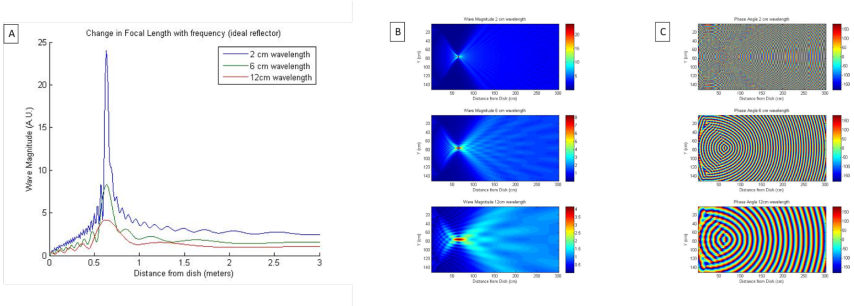

Figure S1: The focal point of the dish does not vary with frequency. In A, we show a 1D slice of Fresnel-diffraction simulations of an ideal source being focused onto a focal plane after reflecting off of a discretized reflector at 3 different wavelengths (2cm, 6cm, and 12cm). The deviation between the focal points is minimal. In B, we show the magnitude of the wave across 2 spatial dimensions. In C, we show the phase of the wave across 2 spatial dimensions.

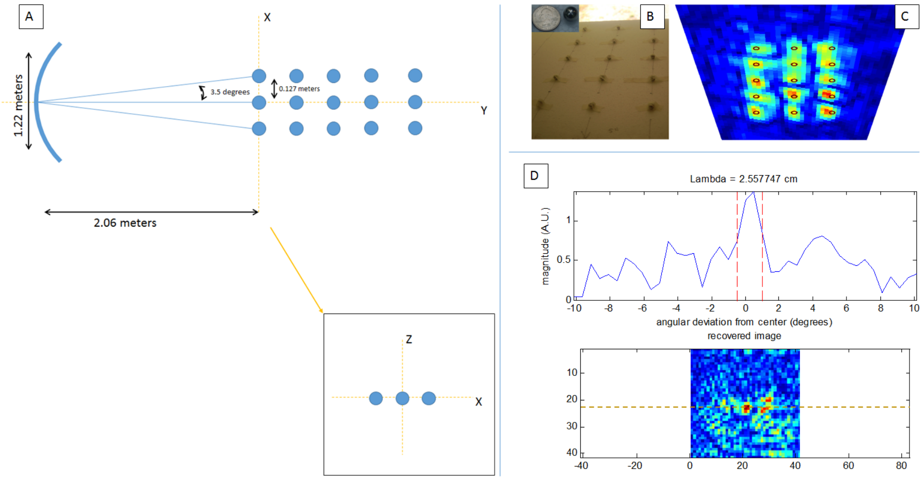

Figure S2: The system has an experimentally measured PSF of 1.5 degrees. A: a depiction of the experiment to determine the depth and angular accuracy of the system. B: The set of point radius metal point targets. C: A depth image of the same targets, red circles at the correct position are overlaid on top of the recovered and rectified image. D: A 1D slice in X of the front row of point targets, showing the width of the PSF of the system.

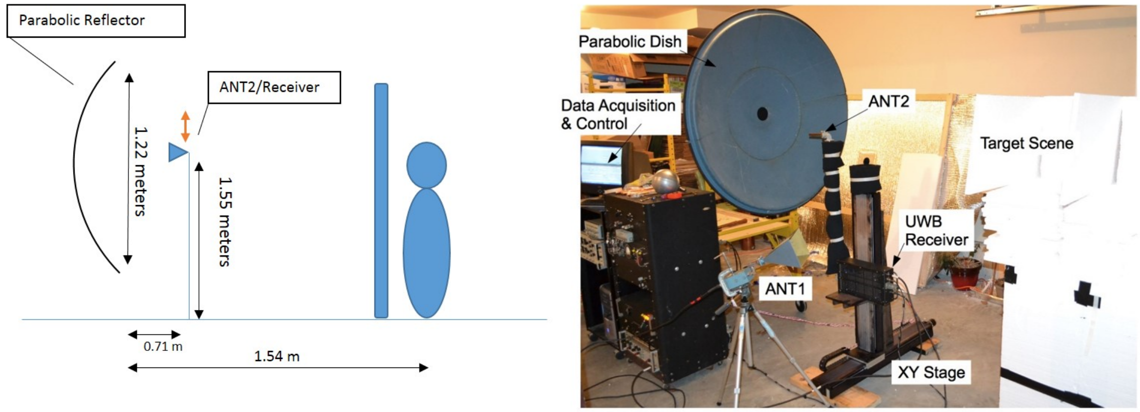

Figure S3: Experimental setup.

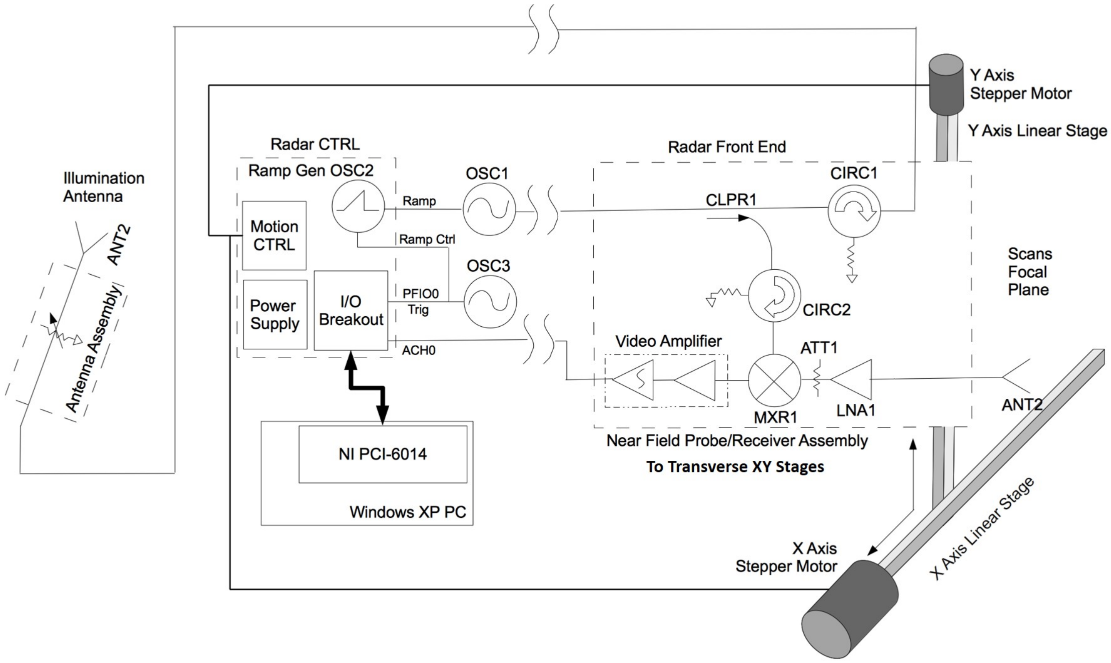

Figure S4: Block diagram.

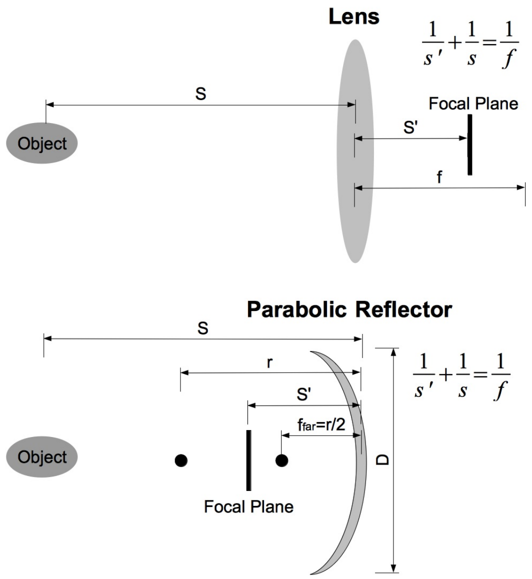

Figure S5: When placing a 2D detector at the focal plane between the radius and focal point of a parabolic reflector the parabolic reflector can be used as a lens.

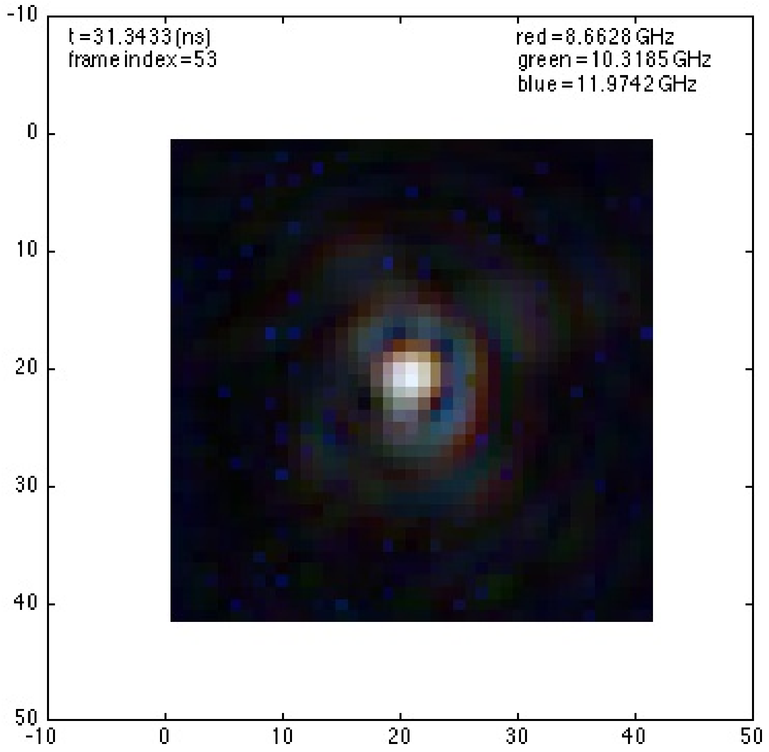

Figure S6: PSF of microwave system. PSF is measured by taking an image of a 15.2mm diameter metal sphere. The sphere reflects the transmitter at a single point. A white-balanced color microwave image of a sphere (calibration procedure explained above in supplementary text).

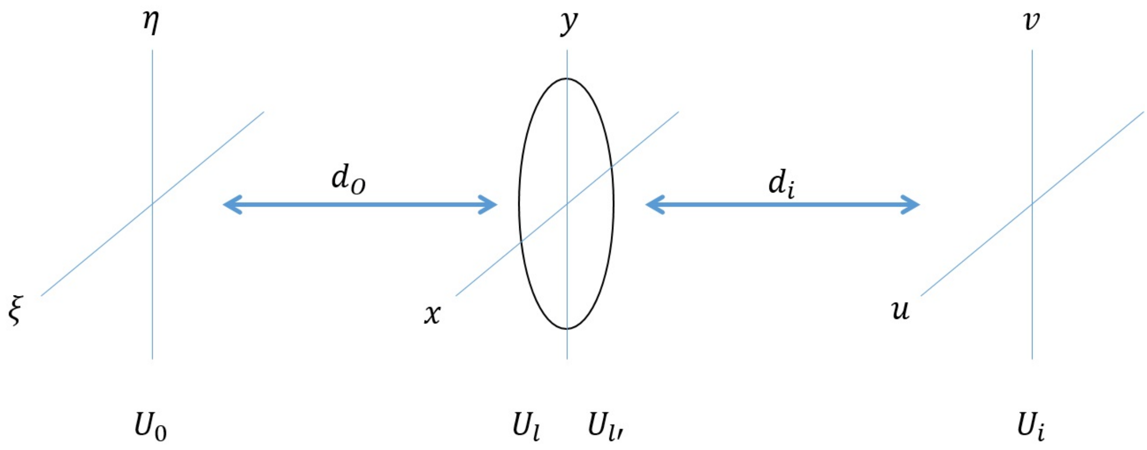

Figure S7: Axes for Fresnel diffraction derivation

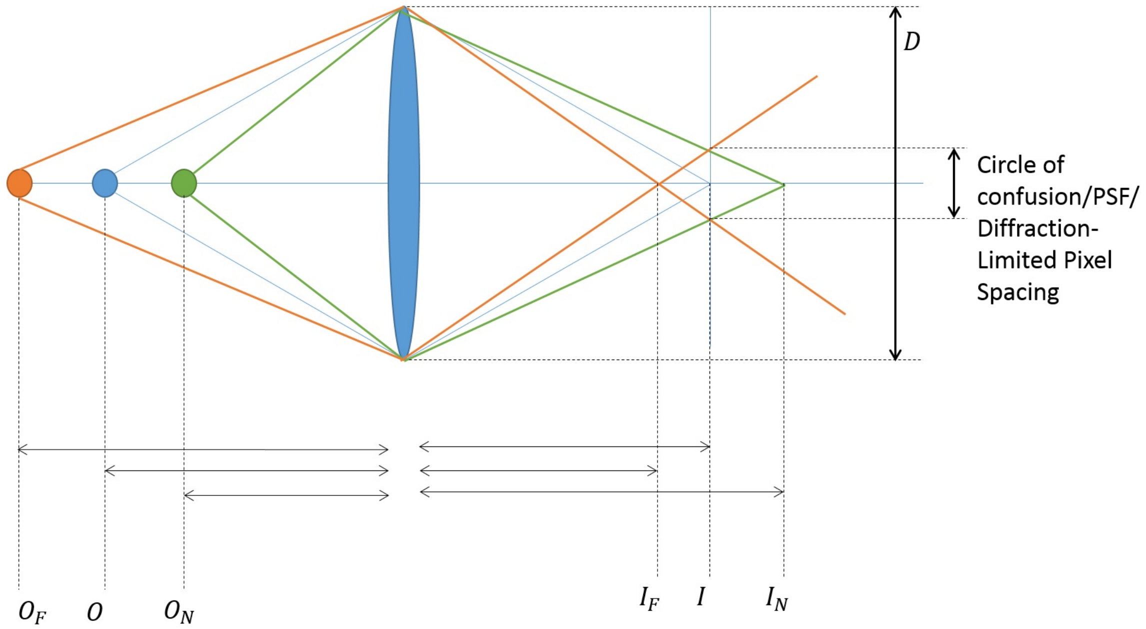

Figure S8: Depth of Field diagram

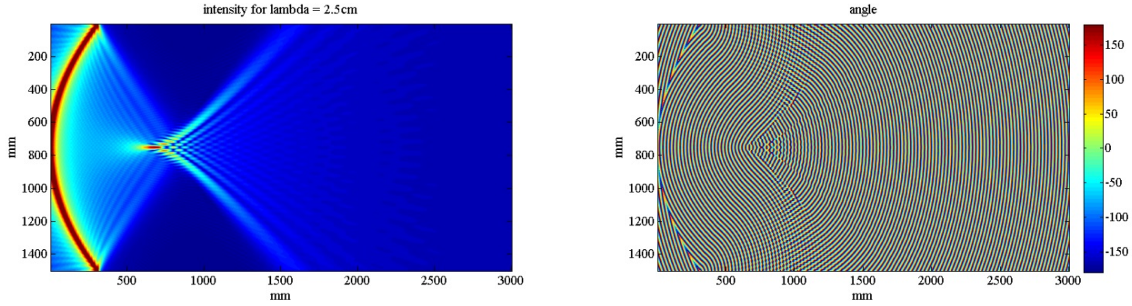

Figure S9: Example of Fresnel-Kirchhoff diffraction simulation. A single point (not shown) illuminates a parabolic reflector which is approximated by diffracting point sources. The waves are focused to a point in front of the reflector. Note that this is a coherent simulation at a single frequency.

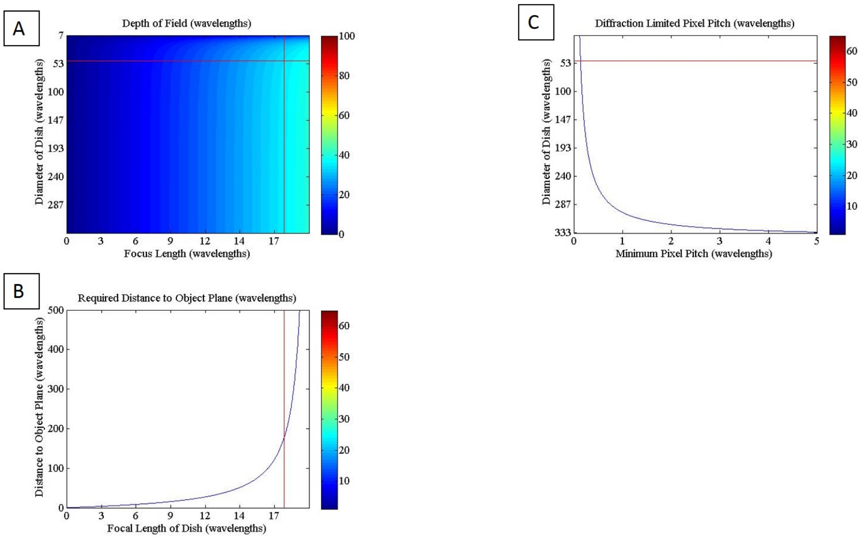

Figure S10: Selecting system geometry parameters such as dish diameter and focal length affect the system depth of field. The DOF for the camera design space is shown in (**A**). In order to increase depth of field, either the diameter or the focal length of the reflector can be increased. Increasing the diameter of the dish also increases the minimum pixel pitch (**C**), thus increasing the size of the focal plane array. By increasing the focal length of the dish, the object distance from the camera must be increased (**B**). The red lines here signify the operating characteristics of our camera.

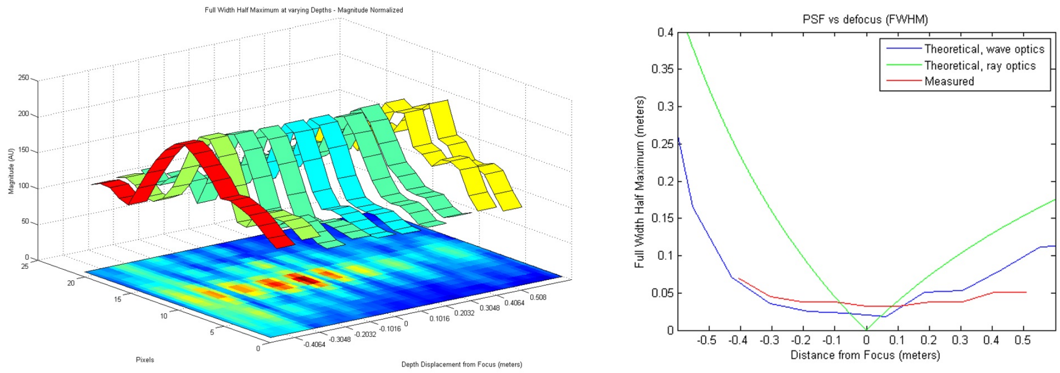

Figure S11: The PSF of Out of Focus (OOF) reflectors is larger than in-focus reflectors. Here the theoretical-ray, theoretical-wave, and experimentally-measured PSF of out of focus elements is shown. The experimentally-measured curve was found by placing reflectors displaced from the focal point and measuring their full width half maximum. Note that the ray-optics perspective approaches an infinitesimally small focus point, while the wave-optics curve is diffraction limited. The theoretical ray-optics curve is shifted to correct for the near-field aberration caused by the parabolic reflector so that the focal point of the ray-optics perspective from the lens equation matches the wave-optics focal point.

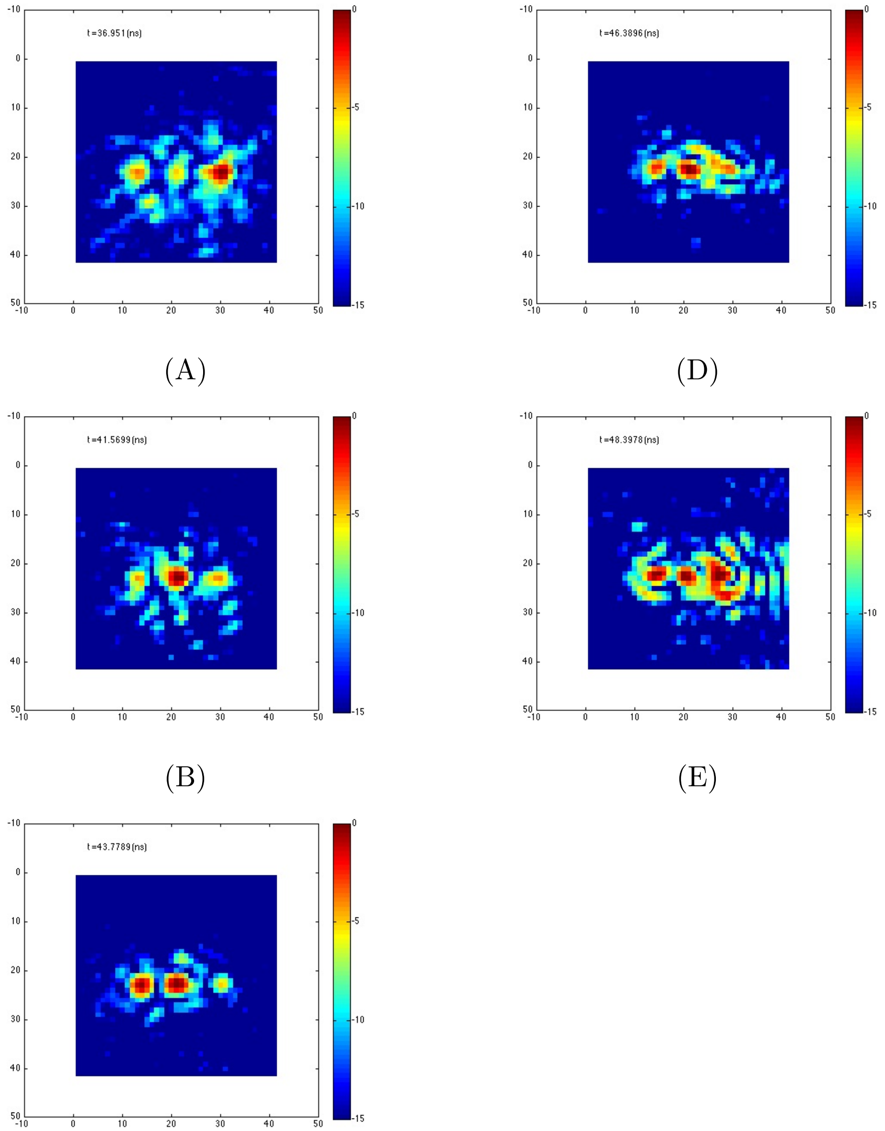

(A) (D)

(B) (E)

(C)

Figure S12: A fly-through of the grid of 0.5 inch diameter spheres, where although of equal spacing throughout the scene, the three spheres appear to be getting closer and closer to each other as time progresses (**A-E**). This is due to the camera projection. This is intuitive if one were to contemplate how railroad tracks converge to a vanishing point as they travel into the distance as viewed by the eye.

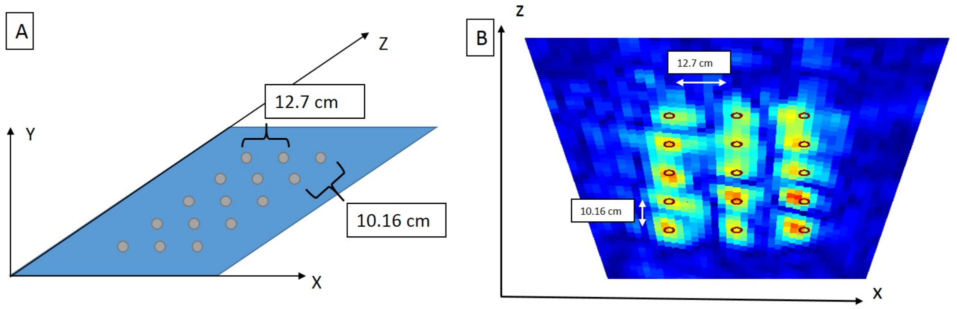

Figure S13: Demonstration of the depth capabilities of the microwave time-of-flight camera. A grid of 12.7 mm reflecting metal spheres are placed in an evenly spaced 5 by 3 grid and imaged edge-on. A schematic of the target is shown in (**A**). The recovered image in (**B**) shows a depth slice of the scene corrected for transmitter location and the camera projection. Red-circles overlaid in the image show the correct location of the spheres. The system is able to recover the position and distribution of the spheres.

Parameter	Presented	Hunt et al	Spinoulas et al	Cull et al	Ghasr et al	Savelyev et al	Equivalent Phased Array	Lens-Based	Lensless
Angular Resolution (degrees)	0.5	1.7	NL	NA	~ 5	< 3.3	0.5	$1.22\lambda/D$	$1.22\lambda/D$
Time-equivalent Resolution (Rayleigh limited) (ps)	200	153	NA	67	NL	100	1/BW	1/BW	1/BW
Number of Sensing Elements	1681 (41 x 41)	101	600	9745 - 21,504 (128 x 128)	576 (24 x 24)	5440 (16 x 340)	7472	N	N
Bandwidth (GHz)	8–12	18.5–25	146–154	94	24	10–18	BW	BW	BW
Object Distance (meters)	1.70	1.5–4	NL	0.048–0.205	0.39	0.5	NA	d_o	d_o
Sensor distance to aperture (meters)	0.46	NA	NL	NA	NA	NA	NA	d_i	NA
Aperture Size (meters)	1.22 x 1.22	0.40 x 0 (1D)	NL	0.30 x 0.30	0.15 x 0.15	0.50 x 0.50	1.22 x 1.22	D	D
Element Pitch (mm)	6.35	NL	NL	2.54	6.30	25	12.5	$d_i \tan(FOV/2)/N$	D/N
Field of View (degrees)	20.25	70	NL	180	180	180	180	As Desired	180
Ratio of Main-lobe to Side-lobe	7.5598	NL	NL	NA	NL	1.99	1	$2J_1(5.13562)/5.13562$	1
Depth of Field (meters)	1.76	∞	NL	∞	∞	∞	∞	[See Above (DoF)]	∞
Focal Length (meters)	0.44	NA	NL	NA	NA	NA	NA	f	NA
Scan Time (seconds)	3600	0.1	NL	1680	0.0455	8280	system dependent	NA	system dependent

Table S1: Common metrics for evaluating a camera are shown above for the microwave camera presented along with values or equations for both lens-based and lenless cameras. Here it is demonstrated how a lens/reflector-based imaging system has advantages over a lensless system since it is possible to achieve a large aperture and a reduced element count while avoiding aliasing and ghosts in images [28, 25]. NA implies the metric is not-applicable. NL implies the metric was not listed in the publication. N is the number of measurements, D is the diameter of the aperture, d_o is the object distance, d_i is the imaging distance, BW is the bandwidth of the system, and λ is the wavelength of transmission. Ranges are specified for papers wherein multiple sparse configurations were used.

References

[1] Skolnik, M. I. Introduction to radar. *Radar Handbook* 2 (1962).

[2] Farr, T. G. *et al.* The shuttle radar topography mission. *Reviews of geophysics* **45** (2007).

[3] Erickson, N. *et al.* A 15 element focal plane array for 100 ghz. *Microwave Theory and Techniques, IEEE Transactions on* **40**, 1–11 (1992).

[4] Erickson, N., Grosslein, R., Erickson, R. & Weinreb, S. A cryogenic focal plane array for 85-115 ghz using mmic preamplifiers. *Microwave Theory and Techniques, IEEE Transactions on* **47**, 2212–2219 (1999).

[5] Goldsmith, P. Focal plane arrays for millimeter-wavelength astronomy. In *Microwave Symposium Digest, 1992., IEEE MTT-S International*, 1255–1258 vol.3 (1992).

[6] Shirokoff, E. *et al.* The south pole telescope sz-receiver detectors. *Applied Superconductivity, IEEE Transactions on* **19**, 517–519 (2009).

[7] Sandri, M., Villa, F. & Mandolesi, N. A view on the planck lfi optics. In *Antennas and Propagation (EuCAP), 2010 Proceedings of the Fourth European Conference on*, 1–5 (2010).

[8] Bonetti, J. *et al.* Transition edge sensor focal plane arrays for the bicep2, keck, and spider cmb polarimeters. *Applied Superconductivity, IEEE Transactions on* **21**, 219–222 (2011).

[9] Yassin, G., Leech, J., Tan, B. & Kittara, P. Easy to fabricate feeds for astronomical receivers. In *Antenna Technology (iWAT), 2013 International Workshop on*, 15–18 (2013).

[10] Locke, L. S., Bornemann, J. & Claude, S. Novel k-band prime focus reflector-coupled focal plane array. In *Microwave Conference (EuMC), 2013 European*, 211–214 (2013).

[11] Rodriguez-Morales, F. *et al.* A terahertz focal plane array using heb superconducting mixers and mmic if amplifiers. *Microwave and Wireless Components Letters, IEEE* **15**, 199–201 (2005).

[12] Tang, A. *et al.* A 200 ghz 16-pixel focal plane array imager using cmos super regenerative receivers with quench synchronization. In *Microwave Symposium Digest (MTT), 2012 IEEE MTT-S International*, 1–3 (2012).

[13] De Jonge, C. *et al.* Development of a passive stand-off imager using mkid technology for security and biomedical applications. In *Infrared, Millimeter, and Terahertz Waves (IRMMW-THz), 2012 37th International Conference on*, 1–2 (2012).

[14] Adib, F. & Katabi, D. See through walls with wifi! In *Proceedings of the ACM SIGCOMM 2013 Conference on SIGCOMM*, SIGCOMM '13, 75–86 (ACM, New York, NY, USA, 2013).

[15] Adib, F., Kabelac, Z., Katabi, D. & Miller, R. C. 3d tracking via body radio reflections. In *11th USENIX Symposium on Networked Systems Design and Implementation (NSDI 14)*, 317–329 (USENIX Association, Seattle, WA, 2014).

[16] Peabody Jr, J. E., Charvat, G. L., Goodwin, J. & Tobias, M. Through-wall imaging radar. *Lincoln Laboratory Journal* **19** (2012).

[17] Ghasr, M., Abou-Khousa, M., Kharkovsky, S., Zoughi, R. & Pommerenke, D. Portable real-time microwave camera at 24 ghz. *Antennas and Propagation, IEEE Transactions on* **60**, 1114–1125 (2012).

[18] Spinoulas, L. *et al.* Optimized compressive sampling for passive millimeter-wave imaging. *Appl. Opt.* **51**, 6335–6342 (2012).

[19] Chan, W. L. *et al.* A single-pixel terahertz imaging system based on compressed sensing. *Applied Physics Letters* **93**, 121105 (2008).

[20] Cull, C. F., Wikner, D. A., Mait, J. N., Mattheiss, M. & Brady, D. J. Millimeter-wave compressive holography. *Applied optics* **49**, E67–E82 (2010).

[21] Hunt, J. *et al.* Metamaterial apertures for computational imaging. *Science* **339**, 310–313 (2013). http://www.sciencemag.org/content/339/6117/310.full.pdf.

[22] Xu, Z. & Lam, E. Y. Image reconstruction using spectroscopic and hyperspectral information for compressive terahertz imaging. *J. Opt. Soc. Am. A* **27**, 1638–1646 (2010).

[23] Charvat, G. L. *Small and Short-Range Radar Systems* (CRC Press, Boca Raton, FL, 2014).

[24] Ruck, G. T., Barrick, D. E., Stuart, W. D. & Krichbaum, C. K. *Radar Cross Section Handbook* (Plenum, New York, NY, 1970).

[25] Goodman, J. *Introduction to Fourier Optics* (Roberts and Company Publishers, 2004), 3rd edition edn.

[26] Levin, A., Fergus, R., Durand, F. & Freeman, W. T. Image and depth from a conventional camera with a coded aperture. *ACM Trans. Graph.* **26** (2007).

[27] Veeraraghavan, A., Raskar, R., Agrawal, A., Mohan, A. & Tumblin, J. Dappled photography: Mask enhanced cameras for heterodyned light fields and coded aperture refocusing. *ACM Transactions on Graphics* **26**, 69 (2007).

[28] Johnson, D. H. & Dudgeon, D. E. *Array Signal Processing: Concepts and Techniques* (Simon & Schuster, 1992).
